# Schaaf-Yang Syndrome: A Real Challenge for Prenatal Diagnosis

**DOI:** 10.7759/cureus.20414

**Published:** 2021-12-14

**Authors:** Sara Nunes, Marta Xavier, Cátia Lourenço, Mónica Melo, Cristina Godinho

**Affiliations:** 1 Obstetrics and Gynecology, Centro Hospitalar de Trás-os-Montes e Alto Douro, Vila Real, PRT; 2 Obstetrics and Gynecology, Centro Hospitalar de Vila Nova de Gaia/Espinho, Vila Nova de Gaia, PRT

**Keywords:** neurocognitive disability, fetal hypotonia, magel2 gene, prader-willi syndrome, schaaf-yang syndrome

## Abstract

Schaaf-Yang syndrome (SYS) is a rare neurodevelopmental disorder caused by pathogenic variants in the MAGEL2 gene. It is usually a postnatal diagnosis in infants with muscular hypotonia and feeding difficulties. There are no cases diagnosed antenatally. During pregnancy, the most common findings reported are polyhydramnios and decreased fetal movements, which are relatively common and unspecific.We present one case of fetal clubfoot and clinodactyly in a fetus postnatally diagnosed with SYS, as well as a brief review of the prenatal findings associated with this syndrome.

## Introduction

Schaaf-Yang syndrome (SYS) is a rare neurodevelopmental disorder, with similarities to Prader-Willi syndrome [1-3]. First described in 2013, it is caused by truncating mutations in the maternally imprinted, paternal copy of the MAGEL2 gene, at 15q11.2q13. This means that only the paternally derived allele is expressed while the maternally one is inactivated. The definitive diagnosis is established either with a whole-exome sequencing test or single-gene sequencing of MAGEL2. Unfortunately, this condition is invariably diagnosed after birth in infants with suspected clinical findings like muscular hypotonia and feeding difficulties [4-7]. To date, there are no published cases of prenatal diagnosis of this syndrome, which is related to the fact that the most frequently reported signs, polyhydramnios and decreased fetal movements, are non-specific findings and can be present in several conditions [5,8-11]. We report one new case of prenatal clubfoot and clinodactyly in a fetus diagnosed postnatally with Schaaf-Yang syndrome.

## Case presentation

**Figure 1:**
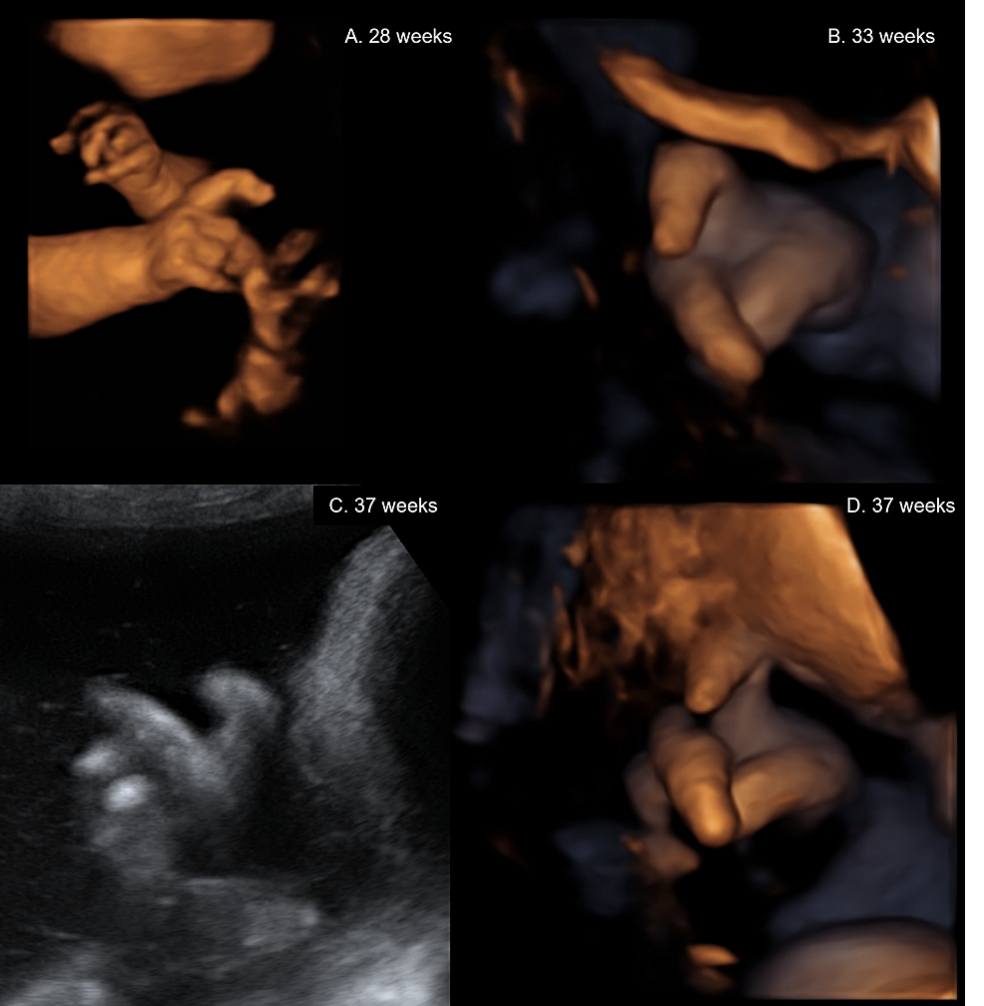
Clinodactyly on 2D and 3D ultrasound at different gestational ages (identified chronologically by letter)

A 37-year-old Caucasian primigravidae was referred to our outpatient obstetric department at nine weeks of gestation by a history of Crohn's disease, well-controlled on bidiary 800 mg of mesalazine. The first-trimester ultrasound performed at 13+5 weeks, with a GE Voluson E8 scanner (GE Healthcare, Austria) and using a 4-8 MHz transabdominal transducer was normal. The combined biochemical screening was negative for aneuploidies. The second-trimester ultrasound, performed on the same equipment, identified bilateral clubfoot in a 20+3 weeks male fetus with no other anatomical anomalies and normal amniotic fluid volume. A fetal echocardiogram, one week later, was normal. Also, array-CGH (obtained by amniocentesis at 21 weeks, according to parents’ decision) was normal. At the 28 weeks' scan, polyhydramnios (amniotic fluid index of 25 cm) and bilateral clinodactyly were detected (Figure 1). Polyhydramnios remained stable until the end of pregnancy and no decreased fetal movements were reported. At 40+6 weeks, a cesarean section was performed after a failed labor induction and a 3 kg male child with an 8/8/10 Apgar score was born. Besides the prenatally diagnosed clubfoot and bilateral clinodactyly, the child displayed global hypotonia and distal arthrogryposis. With these findings and clinical suspicion of a developmental disorder, whole-exome sequencing was requested, and a heterozygotic c.1996dupC MAGL2 gene was detected, thus establishing the diagnosis of Schaaf-Yang syndrome in 2017.

In the first childhood, the boy presented a neurodevelopment delay and disruptive behavior. At the age of seven, he could barely speak but could walk and eat on his own with a spoon. The couple's second child was born seven years after the first, with prenatal single-gene testing negative for SYS.

## Discussion

First described by Schaaf et al. back in 2013 [1], SYS (OMIM 615547, ORPHA 398069) is a rare autosomal dominant neurodevelopmental disease, clinically reassembling Prader-Willi syndrome [2-3]. It is caused by truncating pathogenic variants in the maternally imprinted, paternally expressed MAGEL2 gene [4-6]. This is a heterozygous gene located on chromosome 15 (15q11.2 locus), and only the paternally inherited allele is associated with disease [7]. When the mutated allele is inherited from the father, a future child has a 50% probability of being affected. On the other hand, *de novo* mutations, which comprise 50% of cases, have a 2-3% chance of recurrence. The estimated prevalence is less than 1 per 1,000,000 [8] and there are about 160 cases published in the literature to date [9].

SYS presents with a highly variable, complex phenotype, and the severity of the latter depends on the specific location of MAGEL2 mutation [10]. It is usually diagnosed after birth in infants with global hypotonia, feeding difficulties, contractures, developmental delay, sleep apnea, and/or gastroesophageal reflux [10]. Hyperphagia and excessive weight gain, hypogonadism, short stature, and a unique behavioral profile are commonly seen later during childhood [11-12]. Differential diagnosis includes Prader-Willi syndrome, Chitayat-Hall syndrome, and Freeman-Sheldon syndrome [9]. Though it may be difficult, a timely differential diagnosis between these conditions is fundamental to adequate early treatment and improve prognosis [13]. Joint contractures and/or arthrogryposis (such as in this case) are more suggestive of SYS [3,9]. Hyperphagia is not so prevalent, being reported in only 25% of individuals with SYS [8]. The case presented occurred in the same year that SYS was first described (2013), which justifies the diagnosis delay (accomplished only in 2017). The definitive diagnosis can be determined by genetic testing, through whole-exome sequencing or single-gene sequencing of MAGEL2. In this case, a heterozygotic c.1996dupC MAGL2 gene was confirmed by exome sequencing, which is a well-established pathogenic variant of SYS [4-5]. Mutations of MAGEL2 are generally searched postnatally, in patients with clinically suspicious features that have tested negative for Prader-Willi syndrome [11]. Hypotonia and neurodevelopment delay are very difficult to ascertain antenatally, which explains the difficulty of prenatal diagnosis and the nonexistence of SYS-reported cases, thus proving to be a real challenge. To the best of our knowledge, there are no published cases of prenatal diagnosis of SYS. The sparse data available results from a retrospective evaluation of clinical reports after diagnosis during infancy, and antenatal information is frequently missing. The main prenatal findings reported are polyhydramnios and decreased fetal movements [4,8-11]. Though weight evaluation is generally reported in child and adult phenotypes, we couldn’t find any references to the fetal growth pattern. A review of prenatal findings in patients diagnosed with SYS after birth is presented in Table 1.

**Table 1:** Review of prenatal findings in patients diagnosed with SYS after birth NS = Information not specified or otherwise unavailable; SYS = Schaaf-Yang syndrome Normal weight was defined as birth weight >P10 and <P90 and percentiles were calculated through the Fetal Medicine Foundation birth weight assessment calculator. *Only individuals with molecularly confirmed MAGEL2 mutation and detailed medical history were included in this table.

	n	Polyhydramnios	Decreased fetal movements	Other prenatal findings	Birth weight
Present case	1	Yes	No	Bilateral clubfoot; Clinodactyly	Low for Gestational Age (1)
Marbach (2020) [8]	8	NS	Yes (1/8)	NS	Normal
Andrade (2020) [9]	1	NS	NS	NS	Normal
Negishi (2019) [3]	6	NS	NS	NS	Low for gestational age (2/6) Normal (4/6)
McCarthy (2018) [10]	78	NS	NS	NS	NS
Fountain (2017) [11]	18	NS	Yes (6/15)*	NS	NS
Enya (2017) [6]	3	NS	NS	NS	Low for gestational age (1/3) Normal (1/3)
Mejlachowicz (2015) [5]	4	Yes (3/4)	Yes (4/4)	Unilateral clubfoot (1/4) Bilateral clubfoot (1/4) Bilateral camptodactyly (1/4)	NS
Soden (2014) [4]	2	NS	Yes (2/2)	NS	NS

We present a new case of prenatal bilateral clubfoot and clinodactyly on a fetus diagnosed postnatally with SYS. Mejlachowicz et al. have previously described two cases of prenatal polyhydramnios and decreased fetal movements associated with unilateral and bilateral clubfoot, but in both cases, fetal demise occurred before 25 weeks [5]. On pathological autopsy, camptodactyly was observed in both fetuses. The same author described a third case with a similar phenotype but specific features are not mentioned. Antenatal clinodactyly, described in our case, seems to agree with the hand’s phenotypes described by Fountain et al. [11], which include interphalangeal joint contractures, camptodactyly, tapering of the fingers, brachydactyly, clinodactyly, and adducted thumbs. The skeletal anomalies, like clubfoot, in this case, can suggest the presence of contractures and arthrogryposis commonly associated with SYS [5-6,12]. However, these findings are nonspecific and could also be found in several other congenital conditions. Thus, SYS should be considered a differential diagnosis in the presence of fetal skeletal anomalies and increased amniotic fluid volume, with or without decreased fetal movements.

## Conclusions

SYS is a rare neurodevelopmental disease with nonspecific antenatal findings. Bilateral clubfoot, clinodactyly and polyhydramnios were the prenatal findings in the case reported here. In the case presented by the authors, fetal movements were consistently described as normal by the mother. The arthrogryposis presented by this child immediately after birth, in addition to the prenatal findings, raised a greater degree of suspicion of SYS. The rarity of this genetic syndrome (only 160 cases published in the literature) and its nonspecific prenatal findings make it difficult to establish clear indications to test for fetal MAGEL2 mutations. Further studies are needed to identify which ultrasound and clinical features should raise the hypothesis of SYS during the prenatal period.
